# Analysis of a “3-(Naphthalen-1-ylimino)indolin-2-one” Compound and Its Antimicrobial Assessment Using Lipid-Based Self-Nanoemulsifying Formulations

**DOI:** 10.3390/molecules26010015

**Published:** 2020-12-22

**Authors:** Saeed Ali Syed, Ahmed Bari, Mohammed S. Aldughaim, Md Abdur Rashid, Mohammad Hossain Shariare, Mohsin Kazi

**Affiliations:** 1 Department of Pharmaceutical Chemistry, College of Pharmacy, King Saud University, Riyadh 11451, Saudi Arabia; sasyed@KSU.EDU.SA (S.A.S.); abari@ksu.edu.sa (A.B.); 2 Central Laboratory, Research Center, College of Pharmacy, King Saud University, Riyadh 11451, Saudi Arabia; 3 Research Centre, King Fahad Medical City, Riyadh 11525, Saudi Arabia; maldughaim@kfmc.med.sa; 4 Department of Pharmaceutics, College of Pharmacy, King Khalid University, Abha 62529, Saudi Arabia; mdrashid@kku.edu.sa; 5 Department of Pharmaceutical Sciences, North South University, Dhaka 1229, Bangladesh; mohammad.shariare@northsouth.edu; 6 Department of Pharmaceutics, College of Pharmacy, King Saud University, Riyadh 11451, Saudi Arabia

**Keywords:** UPLC systems, molecule synthesis, method validation, 3-(Naphthalen-1-ylimino)indolin-2-one, antimicrobial assessment

## Abstract

In recent years, indole derivatives have acquired conspicuous significance due to their wide spectrum of biological activities—antibacterial, antiviral, and anticonvulsant. This compound is derived from naturally grown plants. Therefore, synthesis of a novel “3-(Naphthalen-1-ylimino)indolin-2-one” compound (**2**) and its analysis using UPLC systems along with antimicrobial assessment was the aim of the current study. Isatin was used as a parent drug for synthesizing compound (**2**). Liquid Chromatographic analysis was performed using a C18 BEH column (1.7 μm 2.1 × 50 mm) by UPLC systems. Degradation studies were carried out to see whether acid, base, thermal, and oxidizing agents had any impact on the synthesized molecule in stress conditions (100 °C). A lipid-based self-nanoemulsifying formulation was developed and selectivity, specificity, recovery, accuracy, and precision were measured as part of the UPLC system’s validation process. Antimicrobial studies were conducted using gram-positive and gram-negative bacteria. The standard samples were run with a concentration range of 5.0–100.0 μg/mL using the isocratic mobile phase comprising of methanol/water (70/30 %*v*/*v*) at 234 nm; good linearity (R^2^ = 0.9998) was found. The lower limits of detection (LOD) and quantitation (LOQ) of the method were found to be 0.81 μg/mL and 2.5 μg/mL, respectively. The coefficients of variation were found to be less than 2%. The antimicrobial study suggests that compound (**2**) has a substantial growth effect against gram-negative bacteria. It was successfully synthesized and applied to measure the concentrations in lipid-based dosage form, along with potent antimicrobial activities.

## 1. Introduction

**Figure 1:**
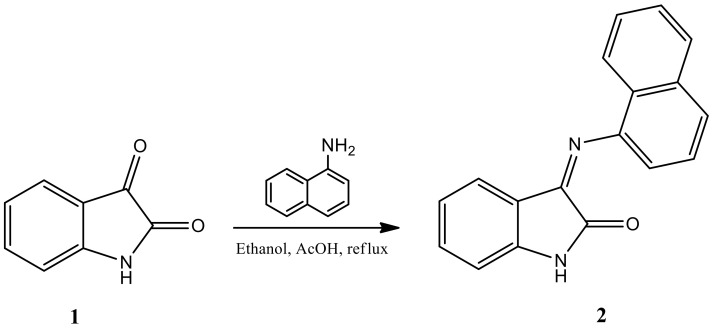
Synthesis of the new molecule “3-(Naphthalen-1-ylimino)indolin-2-one” (**2**) from the parent compound isatin (**1**).

3-(Naphthalen-1-ylimino)indolin-2-one is a compound freshly synthesized in current studies and has been derived from the known compound “1*H*-indole-2, 3-dion” (isatin) (Figure 1). Isatin was first obtained by Erdman and Laurent in 1841 as a product of the oxidation of indigo dye by nitric acid and chromic acids [1,2]. The compound exists in many naturally grown plants. In recent years, indole derivatives have acquired conspicuous significance due to their wide spectrum of biological activities. The synthetic versatility of isatin has led to its extensive use as an antibacterial [3,4] and antiviral agent [5,6]. However, isatin and its derivatives may also possess anticonvulsant activity [7]. It has been noted that isatin and its 3,3-bis (4-amino-2,5-dimethoxyphenyl)-1,3-dihydroindol-3-one derivative has been synthesized by the reaction of isatin and 2,5-dimethoxyaniline and has been evaluated for antioxidant activity [8,9,10]. In addition, the antiviral activity of isatin and its derivatives have been studied before [11,12,13]. It is worth mentioning that over nearly three decades, many research groups have emerged to search for new anti-infection agents in the field of ethnopharmacology. The anti-inflammatory activity of isatin has also been reported in various studies [14,15,16]. Due to the various medicinal activities of isatin and its derivatives, the compound was freshly synthesized by the indoline-2,3-dione reflux method using absolute ethanol, acetic acid (AcOH) and Naphthalen-1-amine to obtain compound (**2**). This compound has been well-reported in the literature for its biological activities [17,18]. This derivative of isatin could be a potential antiviral and or antibacterial drug molecule for future treatment.

This is the reported compound; however, we synthesized it in our lab and no analytical method had been reported before on the determination and qualitative analysis of “3-(Naphthalen-1-ylimino)indolin-2-one” using UPLC systems. In addition, no liquid chromatographic method is currently available for the qualitative and quantitative analysis of compound (**2**). It is not a commercial product nor is it available in various dosage forms. However, this newly developed analytical method can be utilized for future market products.

Therefore, the objective of the current study was to develop a sensitive UPLC method to identify and quantitate the freshly synthesized molecule “3-(Naphthalen-1-ylimino)indolin-2-one” in order to develop self-nanoemulsifying lipid-based formulations in a dosage form (capsules/tablets). In addition, compound (**2**) and its parent molecule isatin were evaluated as potential antimicrobial compounds. This study found no interference during the analysis/precise quantitation of the synthesized compound (**2**) with the parent compound, isatin. Therefore, the UPLC developed method is new, simple, sensitive, accurate, economic, and reproducible. It has also been found that there is no interference with other excipients used in its novel self-nanoemulsifying lipid-based formulations. The experimental method’s development for formulation and stability studies (under forced acidic, alkaline, thermal, and oxidative degradation processes) were carried out based on the standard International conference on Harmonization (ICH) guidelines [19].

## 2. Results and Discussion

### 2.1. NMR and Mass Analysis of 3-(Naphthalen-1-ylimino)indolin-2-one (***2***)

**Figure 2:**
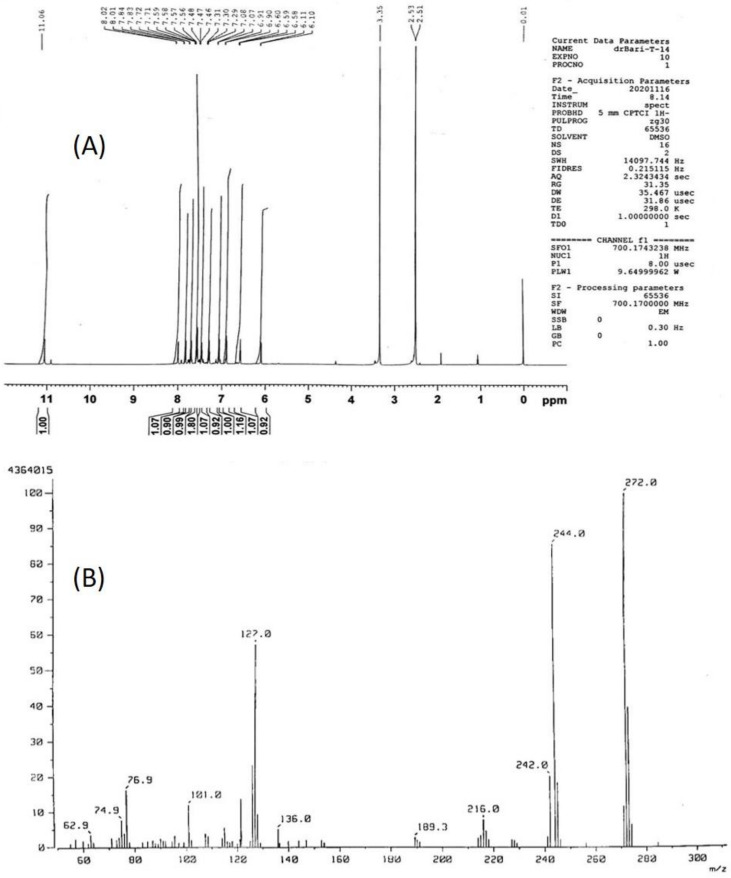
^1^H-NMR spectra (500 MHz) (**A**) and mass spectra (**B**) of 3-(Naphthalen-1-ylimino)indolin-2-one.

In continuation with our research interest, isatin was selected due to its proven anti-tumor and antimicrobial properties. The postulated structure **2** was confirmed by NMR and mass spectrometric data (Figure 2 and in Supplementary Materials) [20]. The results of linear regression showed excellent linearity (approaching a straight-line function) for the model compound (**2**). All the aromatic protons appear in the downfield region of the proton spectra. NH of isatin moiety appears around 11.06 ppm, which confirms the proposed structure. Moreover, mass spectroscopic data is also in accordance with the structure (Figure 2B and in Supplementary Materials). The *m*/*z* 272 is the molecular ion peak, while the fragment around *m*/*z* 244 was due to removal of the carbonyl group. Furthermore, *m*/*z* 144 revealed the molecule of naphthalene along with the postulated structure of compound (**2**).

### 2.2. Optimization of UPLC Peak Separation Conditions

The goal of the developed assay method was peak separation and detection of compound (**2**) molecule without any interference from the parent molecule or relevant substances used in formulation design. Thus, method development was initiated with the optimization of chromatographic conditions, including mobile phase composition. Mobile phase comprising of methanol and water (70/30; %*v*/*v*) was shown to improve signal-to-noise ratio and was found to be suitable for the peak separation of the analyte compound (**2**). The separation with good symmetry was achieved with the Acquity™ UPLC BEH C_18_ (2.1 × 50 mm, 1.7 μm) column with 0.1 mL/minute flow rate and by maintaining the column oven temperature at 35 °C.

**Figure 3:**
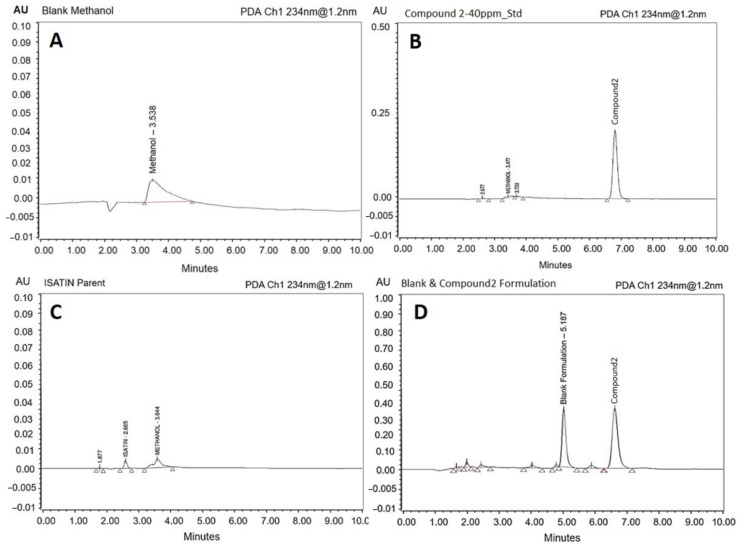
The representative UHPLC chromatograms of the blank methanol sample (**A**), standard solution of compound (**2**) (model drug), conc. 40 μg/mL (**B**), standard solution of isatin (parent drug), conc. 5 μg/mL (**C**) and blank lipid formulation and compound (**2**) containing lipid formulation sample (**D**).

The chromatographic results in the current analysis showed that the compound (**2**) can be determined in the self-nanoemulsifying lipid formulations (SNEDDS) with high sensitivity and selectivity. Figure 3 shows the representative chromatograms of the blank methanol sample (Figure 3A), standard solution of compound (**2**), 40 μg/mL (Figure 3B), parent molecule isatin (Figure 3C) and blank SNEDDS formulation and compound (**2**) containing the lipid-based SNEDDS formulation sample (Figure 3D). Compound (**2**) was well separated from the solvent peak (used as mobile phase and for sample dilutions) with retention time of around 6.8 min (Figure 3B–D). The total chromatographic run time was 10.0 min, where compound (**2**) and isatin peaks were of good shape with full resolution.

### 2.3. Linearity and Range

**Figure 4:**
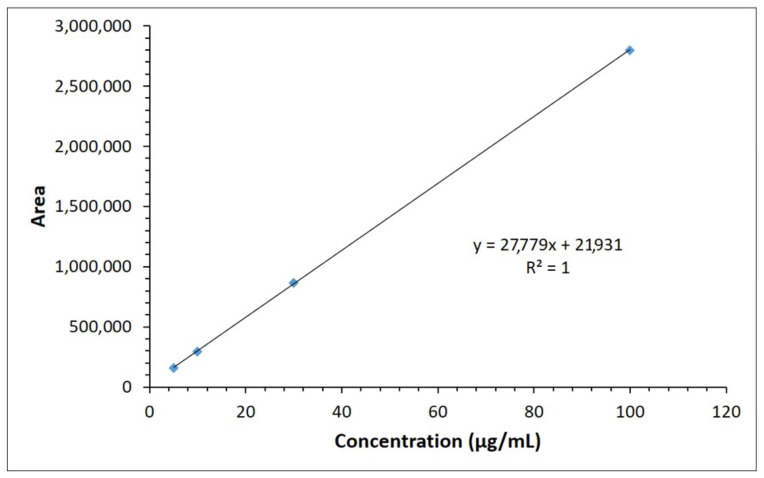
The calibration curve of compound (**2**). The chromatographic conditions used were UPLC Acquity C18, column 1.7 μm (2.1 × 50 mm) maintained at 35 °C temperature, with mobile phase Methanol/water mix. (70/30 %*v*/*v*) at flow rate of 0.1 mL/min. A PDA detector was used at 234 nm wavelength.

A five-point standard calibration curve was constructed separately for compound (**2**) to assess the linearity within the targeted concentration range. The peak area responses were linear over the concentration range between 5.0 and 100 μg/mL (Figure 4). The result of linear regression showed an excellent linearity (approaching a straight-line function) for the drug compound (**2**) over the interval studied. Slope, intercept, correlation coefficient (R^2^), standard deviation of slope, and intercept (obtained by the linear least squares equation of the results) are listed in Table 1. The linearity was evaluated by calculation of the relative standard deviation (RSD) of the slope.

In further confirmation, in the blank chromatogram, a peak was observed at RT 3.507 min, whereas the standard 40 μg/mL solution showed “3-(Naphthalen-1-ylimino)indolin-2-one” (labelled as compound (**2**)) peak eluted at RT 6.821 min. This method can be used for the assay of this molecule in the same formulation. The total run time of the injection was setup to 10 min at a flow rate of 0.1 mL per minute. However, retention time can be further advanced if the flow rate is increased.

### 2.4. System Suitability

System suitability parameters were checked for the developed method using calibration standard solution. Chromatographic parameters such as standard % RSD of solution, Tailing “T”, and theoretical plates “N” were calculated against the peak of “3-(Naphthalen-1-ylimino)indolin-2-one” (compound (**2**)).

**Figure 5:**
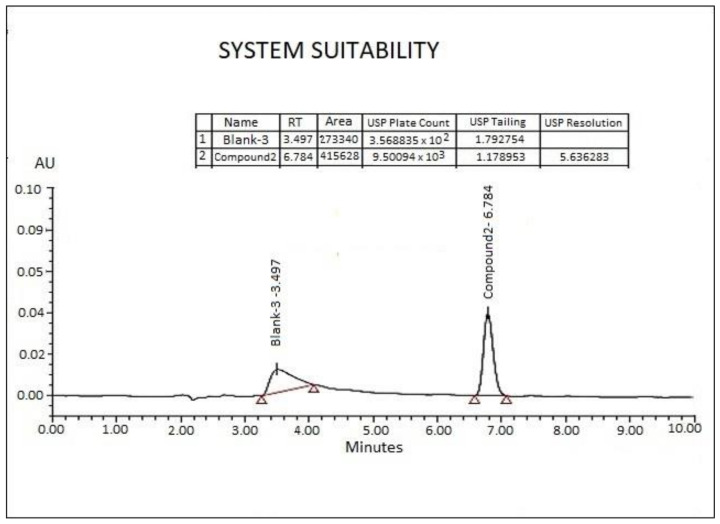
The UPLC chromatogram showing the systems suitability performance of the sample run. Note: * The chromatographic conditions: A Waters Acquity^®^, UPLC C18 BEH column (2.1 × 50 mm, 1.7 μm) kept at 35 °C. The detector wavelength was set at 234 nm for compound (**2**) and isatin, respectively.

The % RSD of the peak area of six consecutive injections of the standard solution was observed to be 0.8%, whereas tailing was observed to be 1.17. The number of theoretical plates (N) was found to be 9500; the overall results reveal that the performance of system is quite acceptable. The parameter of system suitability results is presented in Figure 5. These results of theoretical plates (N) and tailing (T) were calculated by Empower Software (Milford, MA, USA) provided by Waters, used during the UPLC analysis of the samples.

### 2.5. Force Degradation Studies

The force degradation studies were conducted as the solution of molecule “3-(Naphthalen-1-ylimino)indolin-2-one” (compound (**2**)) was subjected separately to thermal degradation, degradation by hydrochloric acid 1N, degradation by sodium hydroxide 1N, and by 3% hydrogen peroxide.

#### 2.5.1. Acid Degradation

**Figure 6:**
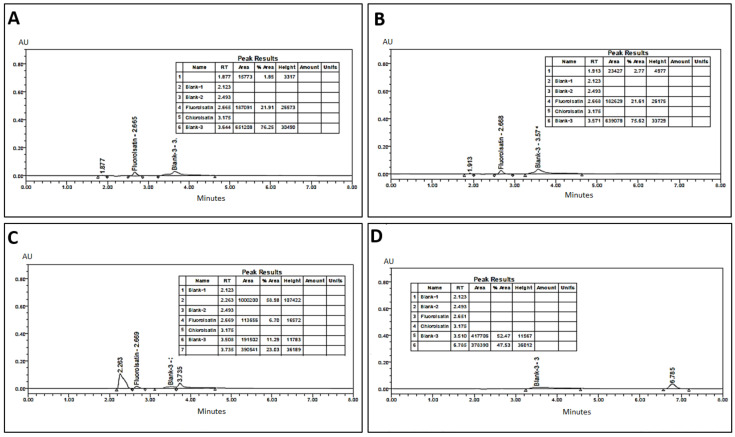
Typical UPLC chromatograms of: (**A**) acid hydrolysis-degraded compound (**2**), (**B**) base hydrolysis-degraded compound (**2**), (**C**) oxidative-degraded compound (**2**) with 3.0% H_2_O_2_, and (**D**) thermal-degraded compound (**2**).

This study was done to investigate the effect of acid degradation on compound (**2**). A 5.0 mL solution of 15 ppm concentration was transferred to a 25 mL volumetric flask, and 5.0 mL of 1N hydrochloric acid was added to this solution and kept for 2 h at 100 °C in a temperature-controlled oven. After 2 h, the sample was taken out from the oven and cooled down to room temperature. Then 5.0 mL of 1N NaOH solution was added to the same flask and diluted to volume with a diluting solvent and subjected to UPLC analysis. The results from Figure 6A show that the peak of compound (**2**) completely disappeared and another two peaks were eluted at RT 1.877 and RT 2.665 min.

#### 2.5.2. Base Degradation

This study was done in context of the acid degradation effect on the molecule, whereas 5.0 mL solution of 15 ppm concentration sample was transferred into a 25 mL volumetric flask. Then, 5.0 mL of 1N NaOH was added to the solution and kept for 2 h at 100 °C in a temperature-controlled oven. After 2 h, the sample was taken out from the oven and allowed to cool down to room temperature. Then 5.0 mL of 1N HCl solution was added to the same flask and diluted to volume with a diluting solvent and subjected to UPLC analysis.

Figure 6B shows almost the same results as shown in Figure 6A (acid degradation). The peak of compound (**2**) in the base degradation test completely disappeared, whereas two peaks were yielded at RT 1.877 and RT 2.665 min.

#### 2.5.3. Oxidation

This study was carried out to evaluate the oxidation effect of hydrogen peroxide, on “3-(Naphthalen-1-ylimino)indolin-2-one”. A 5.0 mL solution of 15.0 ppm standard solution of the sample was transferred to a 25.0 mL volumetric flask and then 5.0 mL of 3.0% hydrogen peroxide solution was added and heated in a water bath at 100 °C for 2 h. The sample was withdrawn from the water bath, cooled down to room temperature, and diluted to volume with diluting solvent methanol and finally subjected to UPLC analysis.

Figure 6C shows that during the oxidation degradation process, the active peak of compound (**2**) completely disappeared. In addition, a new distorted shaped peak appeared at 2.263 min, which indicated that compound (**2**) produced the oxidation product.

#### 2.5.4. Thermal Degradation

A 5.0 mL solution of 15 ppm concentrated standard solution of compound (**2**) was transferred to a 25-mL volumetric flask and kept under controlled temperature at 120 °C in an oven for 24 h. The sample was then diluted to volume with a diluting solvent (methanol) and subjected to UPLC analysis. The chromatogram shown in Figure 6D revealed the thermally degraded products from 15 ppm standard solution. In thermal degradation analysis, the reduction in peak area was observed to be about 10%, as compared to the peak area of actual concentration of 15 μg/mL standard solution.

Overall, the degradation studies suggested that thermal degradation at 120 °C was about 10% reduction in the peak area of the same concentration of solution (15 ppm), whereas in acid and base degradation, the whole compound was degraded (as shown in the chromatogram). However, an unknown peak appeared at 2.66 in both acid and base degradation, while in the case of force oxidation degradation, the main peak was not observed and a presumable oxidation product of compound (**2**) appeared at retention time of 2.263 min.

### 2.6. Accuracy (Recovery) and Precision of “3-(Naphthalen-1-ylimino)indolin-2-one” Compound

#### 2.6.1. Precision

##### Intra-Day

Three concentrations (QC samples) of compound (**2**) (5.0, 10.0, and 100.0 μg/mL) were analyzed three times intra-day. The method developed in the analytical procedures was found to be precise as the intra-day standard deviation (SD) values (Table 2) of six replicate analyses were within the range of 0.019–1.29 μg/mL. Within the analytical concentration range of 5.00–100.0 μg/mL, %CV (coefficient of variation) values were less than 1.246% for compound (**2**). The percentage recoveries were considered good, confirming the repeatability of the current methods.

##### Inter-Day

Three concentrations of compound (**2**) (QC samples—5.0, 10.0, and 100.0 μg/mL) mentioned previously were repeated inter-daily on three different days for the analysis. The inter-day (Table 2) accuracies of six replicates during the three consecutive days were between 99.32% and 99.67%, whereas the %CV values were less than 1.07%. Thus, these low values of both SD and %CV during the intra-day and inter-day analysis met the acceptance criteria of precision for the current method.

### 2.7. Application

**Figure 7:**
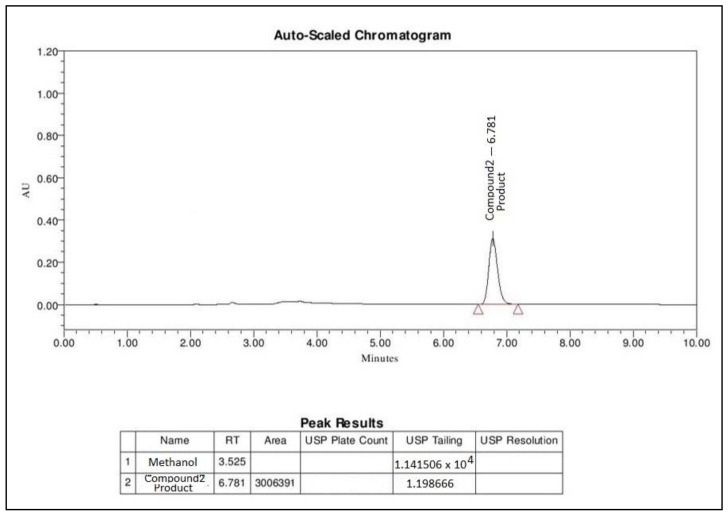
Determination of “3-(Naphthalen-1-ylimino)indolin-2-one” in a lipid-based self-nanoemulsifying formulation (SNEDDS) prepared in-house.

Within the scope of the current analysis, the developed UPLC method was successfully used for the quantification of novel compound, 3-(Naphthalen-1-ylimino)indolin-2-one (compound (**2**)), in studies on equilibrium solubility of self-nanoemulsifying lipid-based formulations (SNEDDS). Our own SNEDDS formulation developed in the lab was used due to an unavailable commercial dosage form of compound (**2**) [21]. A chromatogram of the solubility profile is shown in Figure 7 as an example of method application.

The data represented a concentration of compound (**2**), from a SNEDDS formulation. The SNEDDS formulation consisted of a mixture of long chain triglycerides (black seed oil), medium chain mono- and di-glycerides (Imwitor 988), and a water-soluble non-ionic surfactant (Kolliphor EL) with 5 mg of compound (**2**) as solubilized form. From the results, it can be stated that the current method quantified compound (**2**) precisely (98.2%), which was present in a single unit dosage form (Table 3).

Satisfactory results were obtained in good agreement with the label claimed. The percentage (%) of the labeled claim found from our in-house developed SNEDDS formulation was 98.2% (Table 3). The UPLC chromatogram of compound (**2**) (presented above) was matched with the same retention time of the standard drug molecule.

### 2.8. Antimicrobial Activity Screening

#### 2.8.1. Sample Preparation

10.0 ± 0.1 mg of each sample was taken and diluted with 1.0 mL of dimethyl sulfoxide (DMSO), (Darmstadt, Germany) and was used for the antimicrobial assessments. Isatin and synthesized compound (**2**) molecule powder were loaded into a lipid-based self-nanoemulsifying formulation (SNEDDS) containing black seed oil (BSO), mono- and di-glycerides (Imwitor 988), and non-ionic surfactant Kolliphor EL (castor oil).

#### 2.8.2. Cup-plate Method

A common cup-plate method was utilized for the antimicrobial studies. Isatin as parent compound and the synthesized model compound (**2**) loaded SNEDDS formulations [BSO/Imwitor988/Kolliphor EL (35/15/50, %*w*/*w*)] were used as the dosage form. The pure compound of isatin and compound (**2**) were also used as control. Eight different bacterial strains—*S. aureus*, *E. coli*, *B. subtilis*, *P. aeruginosa*, *A. baumannii*, *Asp. niger*, *Mycobacterium*, and *C. albicans*—were used in the antimicrobial tests. A 100 μL of each sample was added in the cup for carrying out the tests and the results were compared.

#### 2.8.3. Activity Performance

**Figure 8:**
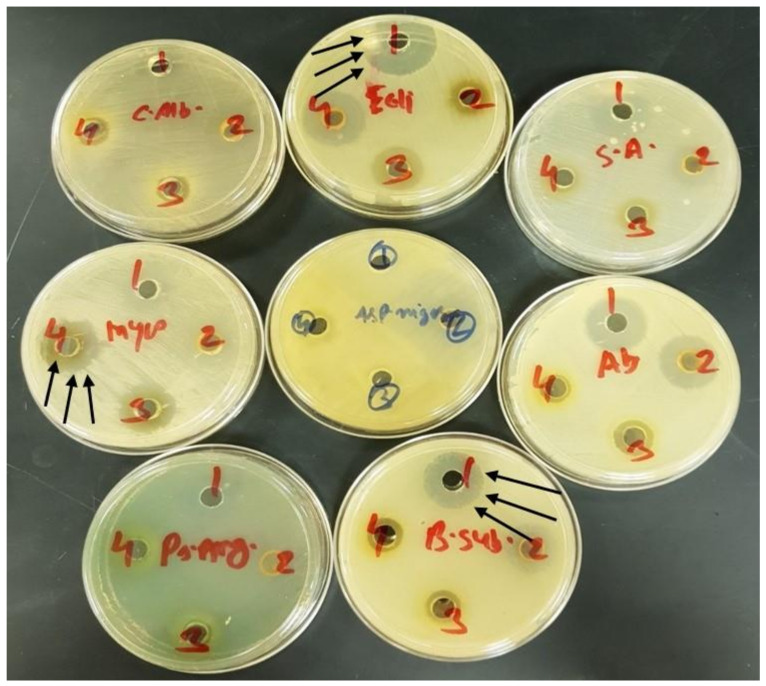
The images show the antimicrobial activities against *S. aureus*, *E. coli, B. subtilis, P. aeruginosa, A. baumannii, Asp. niger, Mycobacterium,* and *C. albicans.* The compounds represent (1) isatin pure drug, (2) compound (**2**) pure drug, (3) isatin loaded lipid-based formulation, and (4) compound (**2**) loaded lipid-based formulation. The arrow indicates the maximum zone of inhibition.

The samples demonstrated a varying level of antibacterial and antifungal activity (shown in Table 4). Isatin and compound (**2**) showed antimicrobial activity against gram-positive bacteria including *Bacillus subtilis* ATCC 10400 and *Staphylococcus aureus* ATCC 29213. On the other hand, both compounds showed better activity against gram-negative bacteria, including E. coli ATCC 25922 and *Acinetobacter baumannii* (clinical strain obtained from the College of Medicine, King Saud University); however, less activity was observed against *Pseudomonas aeruginosa* ATCC 27853. Isatin and synthesized molecule **2** also showed no activity against *Mycobacterium* (clinical strain obtained from the College of Medicine, King Saud University). Isatin and compound (**2**) showed good activity against yeast i.e., *Candida albicans* ATCC 10231 and fungi *Aspergillus niger*, ATCC 16404. Images of the antimicrobial and antifungal activities from the bacterial strains are shown in Figure 8.

Over the last few years, several reports on the synthesis of heterocyclic molecules with anti-inflammatory potential have been published [22,23,24]. Therefore, there is a need to identify the core moiety responsible for such effects and the best possible nano-formulation. The current paper describes the synthesis of isatin moiety with a higher yield along with analytical and nano-formulation studies.

## 3. Materials and Methods

### 3.1. Materials

Isatin (2,3-indolinedione, purity 97%), 1-naphthylamine (purity ≥ 99.0%), absolute ethanol, and acetic acid were supplied by Sigma Aldrich (St. Louis, MO, USA). “3-(Naphthalen-1-ylimino)indolin-2-one” was synthesized in the central laboratory, College of Pharmacy, King Saud University, (Riyadh, Saudi Arabia). High purity Milli-Q water was obtained through a Milli-Q Integral Water Purification System (Millipore, Bedford, MA, USA). The lipid-based self-nanoemulsifying formulation was developed in-house as a single oral dosage form to apply the method. Black seed oil (BSO) was obtained by cold press method from a local vendor. Imwitor 988 (I988) was kindly supplied by Sasol Germany GmbH (Werk Witten, Witten-Germany) and Kolliphor EL was purchased from BASF (Ludwigshafen, Germany). All other reagents were of analytical grade and used without further purification.

### 3.2. Experimental Methods

#### 3.2.1. Procedure of “3-(Naphthalen-1-ylimino)indolin-2-one” Synthesis (**2**)

A mixture containing isatin **1** (1 mmol) and 1-naphthylamine (1.1 mmol) with catalytic amount of acetic acid was heated under reflux and stirring for 3 h in ethanol. After completion of the reaction, as indicated by TLC, the reaction mixture was poured onto crushed ice and the solid separated was filtered under suction, washed with ice-cold water (50 mL), and subsequently dried to afford pure product **2** (compound (**2**)) in good yield.

#### 3.2.2. NMR Analysis of “3-(Naphthalen-1-ylimino)indolin-2-one” (**2**)

Yield 64 %, red solid, IR (KBr): v = cm^−1^. IR (KBr): 1725 (C=O), 3050 (NH). ^1^H-NMR (700.174 MHz, DMSO-*d*_6_): δ = 6.10 (d, 1H, arom), 6.90 (d, 2H, arom), 7.07 (d, 2H, arom), 7.30 (t, 1H, arom), 7.47 (t, 1H, arom), 7.57 (dd, 2H, arom), 7.72 (d, 1H, arom), 7.87 (d, 1H, arom), 8.01 (d, 1H, arom), 11.06 (br s, 1H, NH). ^13^C-NMR (125.76 MHz, DMSO-d6): δ = 112.0, 112.4, 116.2, 122.2, 123.2, 124.1, 125.4, 125.6, 126.6, 126.7, 127.2, 128.5, 134.2, 135.1, 147.2, 147.6, 156.5, 163.7. MS (70 eV): *m*/*z* (Irel, %) 337 (30) [M + H]^+^, 272 (90), 244 (100): calcd for C_18_H_12_N_2_O_2_ (272.09): HRMS: 272.0427 [20].

#### 3.2.3. UHPLC Analysis of “3-(Naphthalen-1-ylimino)indolin-2-one” (**2**)

In UPLC analysis, HPLC grade methanol was used as a diluting solvent for sample preparations (Code M/4056/17 supplied by Fisher Scientific, UK) and in the mobile phase. Milli-Q water was used, which was obtained using the MilliQ-5 Integral Water Purification System (Millipore, Bedford, MA, USA). Waters Acquity™ UPLC systems were used for the analysis, equipped with a PDA detector, binary solvent manager, and auto-sampling manager. The data obtained from the experimental sample run was analyzed by Empower Software 2154 SPs. Analytical balance Shimadzu Library AEG-220 (Shimadzu Corporation Japan), and Bransson 5800 Ultrasonic bath (Branson Corporation, Brookfield, CT, USA) were frequently used in the current studies.

This novel compound (**2**) has not been previously analyzed by UPLC (or any other analytical method); hence, the current developed method is the first analytical method for its precise determination. UPLC is a versatile technique, which is cost- and time-effective, with minimal solvent consumption during running of samples. The newly developed method was validated using parameters such as accuracy, precision and robustness, its limit of detection, and limit of quantification. The intra-day analysis was performed to check the short-term stability of the compound.

#### 3.2.4. UPLC Chromatographic Conditions

A highly sophisticated and rapid technique of Acquity™ UPLC was employed for chromatographic separation. The analytical conditions were optimized in relation to stationary phase BEH C18 and mobile phase of 70% methanol and 30% water mixture. This Acquity™ system (Waters Inc. Bedford, MA, USA) was equipped with a binary solvent manager. The automated sampling manager provided the perfect ease to run the sample in a created sequence. The detection source was a PDA detector with an array of 512 photodiodes and an optical resolution of 1.2 nm. The detector was operated within a range of 190 and 800 nm. The method was developed using C18, 1.7 μm (2.1 × 50 mm) with isocratic mobile phase of Methanol/water (70/30 %*v*/*v*) and flow rate of 0.1 mL/minute at 35 °C temperature. The 234 nm wavelength was set for the determination of the “3-(Naphthalen-1-ylimino)indolin-2-one” model compound. All the sample solutions were filtered using Whatman™ 13 mm PTFE filter to remove any suspended particles from the solution. The sample injection volume of 1 μL was used for the analysis. Methanol (100%) was used as a diluting solvent for the samples.

Preparation of stock solution: All glassware were cleaned and dried in an oven at 105 °C, and finally rinsed with methanol and air dried before use. To prepare the stock solution, 10 mg powder of compound (**2**) was weighed on a calibrated balance and transferred to a 100.0 mL volumetric flask. The solution was then added to 10.0 mL of methanol and sonicated for 1.0 min in a Branson ultrasonic bath, and made up to volume with a diluting solvent to obtain 100 μg/mL of stock solution. The stock solution was diluted to various concentrations: 5.0, 10.0, 30.0, 60.0, and 100.0 μg/mL in methanol. A calibration curve was obtained by plotting the peak area against the above-mentioned concentrations of the targeted molecule, compound (**2**).

#### 3.2.5. Analytical Method Development and Optimization

The purpose of this study was to create a selective isocratic UPLC system for qualitative and quantitative determination of compound (**2**). A range of sample solutions were prepared starting from a low concentration of 5.0 μg/mL to a high concentration of 100 μg/mL. After scanning the sample to optimize wavelength, λ_max_ was observed at 234 nm. The flow rate was set at 0.1 mL/minute and pressure were observed at 4000 psi. The sample volume of 1 μL was taken and sample manager system of auto-sampling was used to run the sample through BEH C18, 1.7 μm (2.1 × 50 mm) column of Waters USA, at 35 °C temperature. The calibration curve was obtained using the aforementioned concentrations of the solution against peak areas.

##### Method Validation

The current developed method was validated in terms of linearity, precision and accuracy, limit of detection LOD, and limit of quantification (LOQ), according to a standard line of method validation, validation of analytical procedures: text and methodology.

##### Linearity and Range

100 ppm stock solution of compound (**2**) was prepared freshly before the desired serial dilutions. The stock solution was use to prepared the five non-zero standards ranging from 5 ppm to 100 ppm. The linear regression and correlation coefficient, y-intercept, and slope of the regression line statistically evaluate the linearity of the results.

##### Specificity

Since the compound was purely synthesized and not formulated, and was in free form, there was no point in interacting with other components. However, the analysis of compound (**2**) was carried out and applied in the lipid-based dosage form (SNEDDS) and further drug-excipients peak interferences were investigated.

##### Accuracy and Precision

Accuracy and precision was evaluated by performing the analysis intra-day and inter-day. The replicates of five different concentrations were run through UPLC on different days for the inter-day assessment. The observed values of peak areas of different days obtained close to each other indicate the precise and accuracy of the method [25].

##### Limit of Detection (LOD)and Limit of Quantification (LOQ)

A lower limit of detection (LOD) was determined by the serial dilution (5 μg/mL to 100 μg/mL) of “3-(Naphthalen-1-ylimino)indolin-2-one” compound from the stock solution, to obtain the signal-to-noise ratio (S/N) of at least 3:1 for LOD and 10:1 for LOQ [26]. They were calculated from the slope of the calibration curve using the following Equation (1):

LOD = 3 × SD/S, and LLOQ = 10 × SD/S.(1)

##### Lipid-Based Self-Nanoemulsifying Formulation Development

Formulation for lipid-based self-nanoemulsifying drug delivery systems (SNEDDS) was developed as a dosage form to load the model drug compound (**2**) and carry out antibacterial activities. Initially, 35% Black seed oil (BSO, long chain triglycerides) and 15% Imwitor 988 (mono- & di-glycerides mix) were mixed as a primary mixture. Then 50% of the primary mixture was vortexed with 50% of Kolliphor EL (non-ionic surfactant of hydrogenated castor oil) to prepare the SNEDDS based on a previously published method [27]. Isatin and compound (**2**) drug powder was loaded as 5 mg/g into the SNEDDS formulation for antimicrobial assessment.

#### 3.2.6. Statistical Analysis

The data was expressed as mean ± standard error of mean (SEM). The significance was determined by applying one-way ANOVA. *P* values < 0.05 were considered significant.

## 4. Conclusions

The current UPLC analytical method is novel, reliable, economic, and reproducible for the newly synthesized compound “3-(Naphthalen-1-ylimino)indolin-2-one”. The developed method is sensitive enough to detect low concentrations down to 0.815 μg/mL in a timely manner with peak elute at ≈6.8 min and flow rate of 0.1 mL per minute. Since we used the pure compound, no interference was caused during the analysis with very low solvent consumption. No significant interferences were found by the formulation excipients, diluents, and or degradation products. This method reveals acceptable precision, accuracy, and appropriate sensitivity, and could be applied to future stability studies on dosage form. The synthesized compound (**2**) could be a potential anti-infective agent due to its potential antimicrobial actions against different bacterial strains tested. Finally, future studies on the mechanisms of action, interactions with antibiotics/antivirals or other synthesized compounds, and their pharmacokinetic profiles should be given high priority.

## Figures and Tables

**Table 1:** Statistical data of regression equation for the determination of new molecule “3-(Naphthalen-1-ylimino)indolin-2-one” (compound (**2**)), generated from the proposed method.

Parameter	“3-(Naphthalen-1-ylimino)indolin-2-one”
Concentration range	5–100 PPM
Intercept (a)	6933.932
Slope (b)	28,067
Correlation coefficient (R)	0.9998
RT ^a^	~6.8 min
*λ_max_*	234 nm
Relative standard deviation of slope	0.8%
Limit of detection (LOD) ^b^	2.470493 μg/mL
Limit of quantification (LOQ) ^c^	0.815263 μg/mL

^a^ Mean of three measurements; ^b^ Limit of detection was estimated at a signal–to–noise ratio of 3; ^c^ Limit of quantification was estimated at a signal–to–noise ratio of 10.

**Table 2:** Evaluation of accuracy and precision of the proposed method for the simultaneous determination of “3-(Naphthalen-1-ylimino)indolin-2-one”, by Inter-day and Intr-day assay.

Assay Type	Amount (μg/mL)		Precision (%)	Accuracy (%)	Drug
	Added	Found ± SD			
*Inter-day*	5	4.98 ± 0.03	0.503	99.63	“3-(Naphthalen-1-ylimino)indolin-2-one”
	10	9.97 ± 0.06	0.563	99.67	
	100	99.32 ± 1.06	1.07	99.32	
*Intra-day*	5	4.97 ± 0.019	0.392	99.47	
	10	9.97 ± 0.059	0.598	99.71	
	100	99.32 ± 1.25	1.246	99.32	

**Table 3:** Determination and % recovery of “3-(Naphthalen-1-ylimino)indolin-2-one” (compound (**2**)) in in-house developed SNEDDS) formulation (*n* = 6). BSO = Black seed oil, I988 = Imwitor 988, KrEL = Kolliphor EL.

Real Sample	Manufacturer	Amount Claimed	Found (mg/mL) ± SD	% of Labelled Claim
BSO/I988/KrEL (35/15/50, %*w*/*w*)	In house, Pharmacy lab, King Saud University	5.00 mg	4.91 ± 0.28 (mg)	98.20

**Table 4:** Antimicrobial activity of the compound against eight (8) different bacterial strains and measurement of the inhibition zone. NZ = No inhibition zone, compound (**2**) = “3-(Naphthalen-1-ylimino)indolin-2-one”, isatin-F = Isatin loaded formulation and compound (**2**) and F = compound (**2**) loaded formulation.

N	Compound	Zone of Inhibition (mm)							
		*B. subtilis*	*S. aureus*	*E. coli*	*P. aeruginosa*	*A. baumannii*	*Mycobacterium*	*C. albicans*	*Asp. niger*
**1**	Isatin	22	18	30	16	22	NZ	15	NZ
**2**	Compound (**2**)	18	15	12	15	20	NZ	10	25
**3**	Isatin-F	12	10	20	NZ	15	NZ	10	13
**4**	Compound (**2**)-F	13	12	23	NZ	12	NZ	12	15

## Supplementary Materials

The following are available online. Mass and NMR spectra of compound (**2**).

## Sample Availability

Samples of the compounds are not available from the authors.
