# Supplementary material for: Analysis of a “3-(Naphthalen-1-ylimino)indolin-2-one” Compound and Its Antimicrobial Assessment Using Lipid-Based Self-Nanoemulsifying Formulations
Source: Molecules. 2020 Dec 22;26(1):15. doi: 10.3390/molecules26010015 (PMC7792971; doi:10.3390/molecules26010015)
Supplement: Supplementary file 1 [file molecules-26-00015-s001.pdf]

[Mass Spectrum]
Data : KSU-DI-EI-R5k-AB-T14001 Date : 14-May-2014 08:57
Sample: -
Note : -
inlet : Direct Ion Mode : EI+
Spectrum Type : Normal Ion [MF-Linear]
RT : 1.40 min Scan# : 28
BP : m/z 271.9845 Int. : 400.00
Output m/z range : 50.0000 to 311.9436 Cut Level : 1.00 %

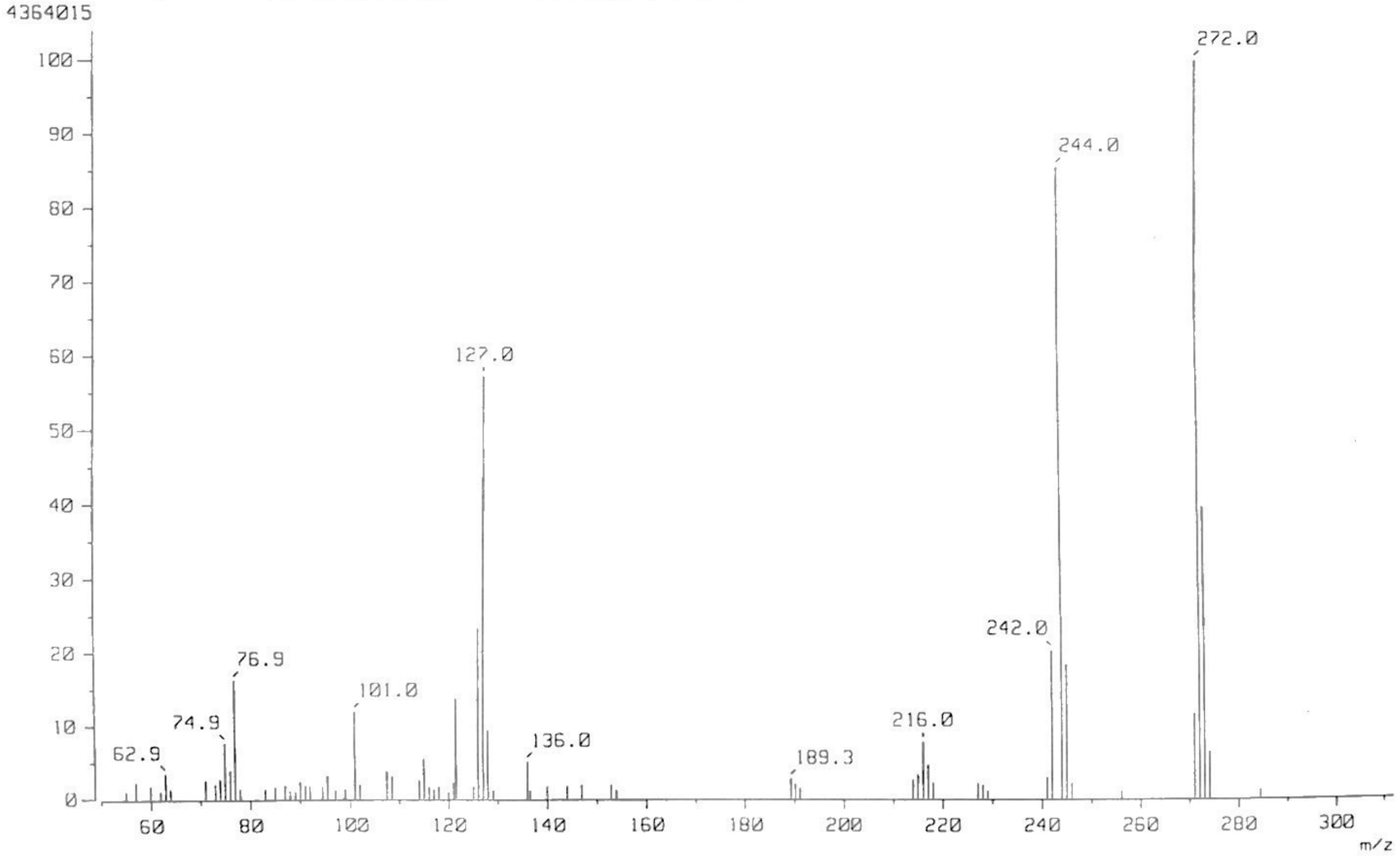

PROTON DMSO {C:\Bruker\TOPSPIN} abari 30

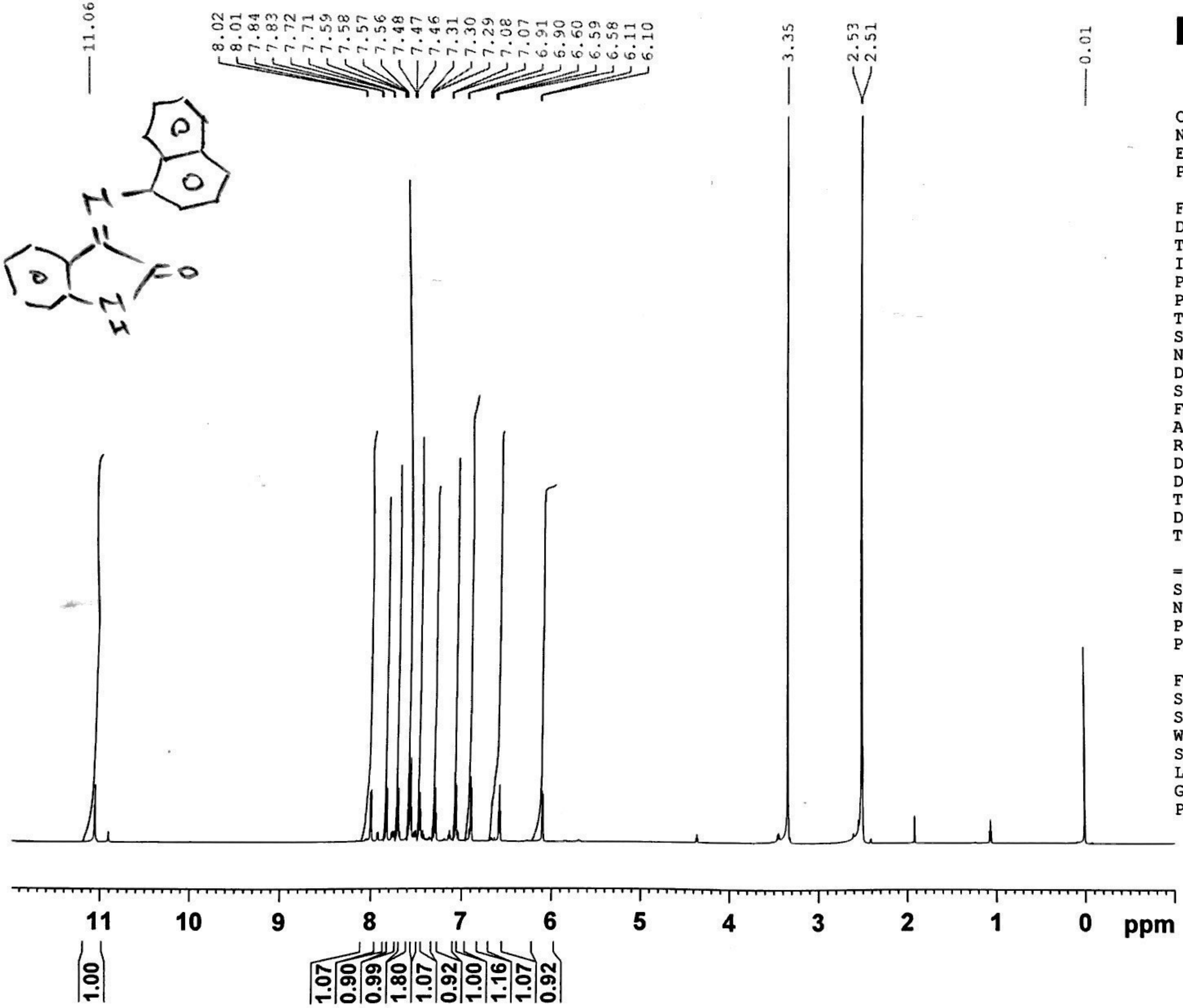

BRUKER

Current Data Parameters

NAME	drBari-T-14
EXPNO	10
PROCNO	1

F2 - Acquisition Parameters

Date_	20201116
Time	8.14
INSTRUM	spect
PROBHD	5 mm CPTCI 1H-
PULPROG	zg30
TD	65536
SOLVENT	DMSO
NS	16
DS	2
SWH	14097.744 Hz
FIDRES	0.215115 Hz
AQ	2.3243434 sec
RG	31.35
DW	35.467 usec
DE	31.86 usec
TE	298.0 K
D1	1.00000000 sec
TD0	1

======== CHANNEL f1 ========

SFO1	700.1743238 MHz
NUC1	1H
P1	8.00 usec
PLW1	9.64999962 W

F2 - Processing parameters

SI	65536
SF	700.1700000 MHz
WDW	EM
SSB	0
LB	0.30 Hz
GB	0
PC	1.00

C13CPD DMSO {C:\Bruker\TOPSPIN} abari 30

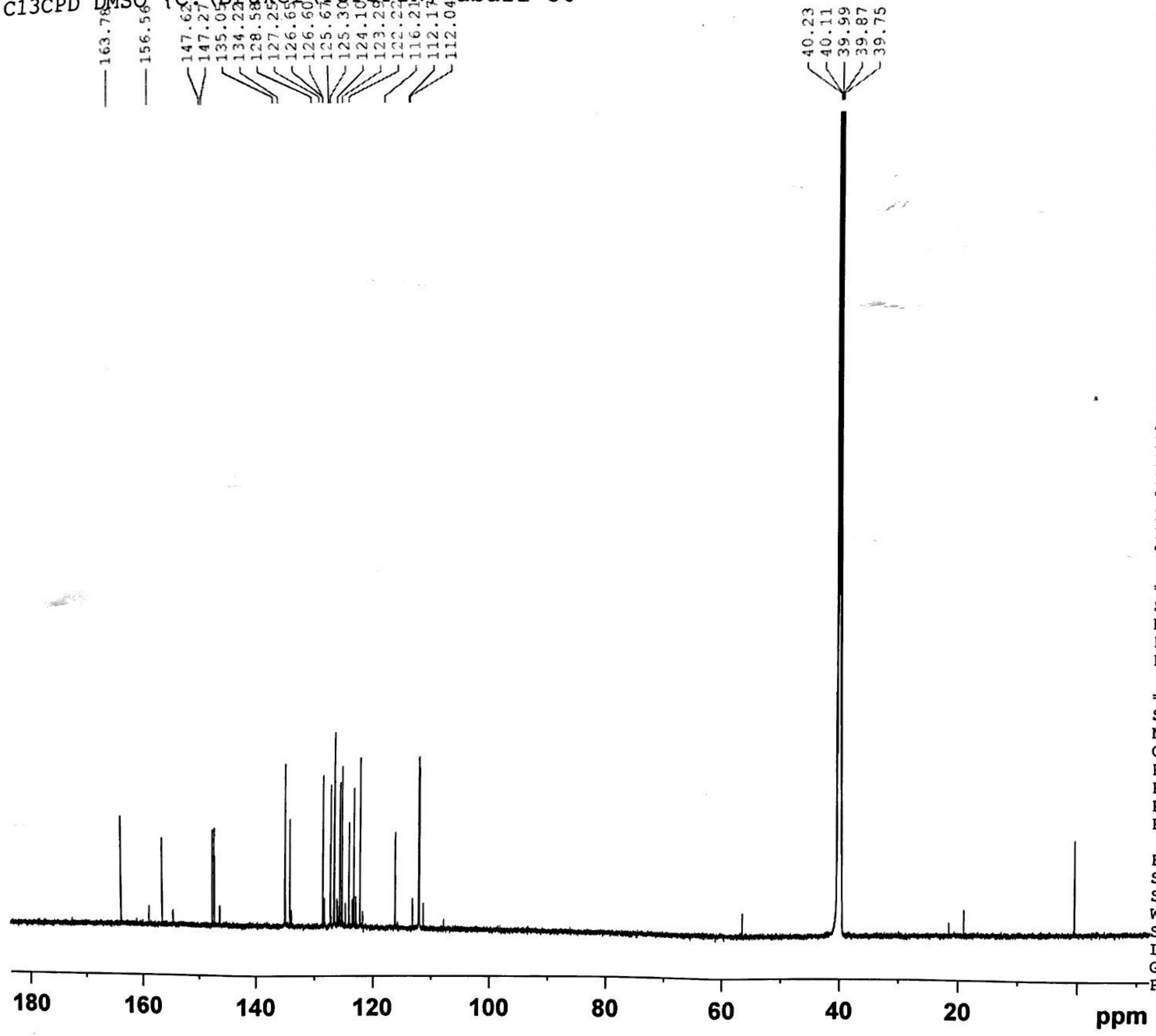

Current Data Parameters	
NAME	drBari-T-14
EXPNO	11
PROCNO	1
F2 - Acquisition Parameters	
Date_	20201116
Time	8.24
INSTRUM	spect
PROBHD	5 mm CPTCI 1H-
PULPROG	zgpg30
TD	65536
SOLVENT	DMSO
NS	739
DS	4
SWH	41666.668 Hz
FIDRES	0.635783 Hz
AQ	0.7864320 sec
RG	172.3
DW	12.000 usec
DE	18.00 usec
TE	298.0 K
D1	2.00000000 sec
D11	0.03000000 sec
TD0	1
======== CHANNEL f1 ========	
SFO1	176.0754915 MHz
NUC1	13C
P1	12.00 usec
PLW1	121.00000000 W
======== CHANNEL f2 ========	
SFO2	700.1728007 MHz
NUC2	1H
CPDPRG[2	waltz16
PCPD2	65.00 usec
PLW2	9.64999962 W
PLW12	0.14618000 W
PLW13	0.06176000 W
F2 - Processing parameters	
SI	32768
SF	176.0578870 MHz
WDW	EM
SSB	0
LB	1.00 Hz
GB	0
PC	1.40
